# Bridging the gap in AMR research: validation of a standardized *Galleria mellonella* model to evaluate *Clostridioides difficile* pathogenesis and treatment efficacy

**DOI:** 10.3389/fphar.2026.1792859

**Published:** 2026-04-13

**Authors:** Faris S. Alnezary, Masaad Saeed Almutairi

**Affiliations:** 1 Department of Pharmacy Practice, College of Pharmacy, Taibah University, Madinah, Saudi Arabia; 2 Department of Pharmacy Practice, College of Pharmacy, Qassim University, Qassim, Saudi Arabia

**Keywords:** antibiotic resistance, Clostridioides difficile, Galleria mellonella, infection model, pathogenesis, vancomycin

## Abstract

**Background:**

*Clostridioides difficile* infection (CDI) is a significant global health burden. *Galleria mellonella* offers an ethical, cost-effective invertebrate alternative to mammalian pre-clinical models. This study evaluated the feasibility of using *Galleria mellonella* to study CDI pathogenesis and treatment.

**Methods:**

We optimized larval storage, temperature, and inoculum size. The model was validated by assessing survival following infection with diverse *C. difficile* ribotypes (RT) and vancomycin treatment. Bacterial burden was quantified via qPCR, and survival was analyzed using Kaplan-Meier curves.

**Results:**

An inoculum of 1 × 10^7^ CFU/mL in fresh larvae (<7 days since receipt) at 37 °C proved optimal. Fresh larvae exhibited significantly higher survival compared to older larvae (P = 0.003). Infection with toxigenic strains resulted in significantly lower survival (35%) compared to uninfected controls (90%; P < 0.001), with virulence varying significantly among RTs. Vancomycin treatment improved survival to 90% in a dose-dependent manner compared to untreated controls (P < 0.001), with a corresponding reduction in bacterial burden confirmed by qPCR.

**Conclusion:**

We conclude that this optimized oral *G. mellonella* model provides a robust and ethically sound high-throughput platform for screening novel anti-*C. difficile* compounds and for comparative virulence studies of diverse ribotypes, effectively bridging *in vitro* assays and mammalian studies for future drug development.

## Introduction

*Clostridioides difficile* infection (CDI) is an urgent public health threat worldwide and is the most common healthcare-associated infection (HAI) in the United States (US) (Guh et al., 2020; Lessa et al., 2015; Feuerstadt et al., 2023). *C*. *difficile*, a spore-forming, anaerobic, toxin-producing bacteria, causes a gastrointestinal infection that manifests with symptoms ranging from mild diarrhea to pseudomembranous colitis (Guh et al., 2020; Salvati et al., 2024). When CDI progresses to toxic megacolon, death may occur in up to 45% of cases (Lessa et al., 2015; Legenza et al., 2018; Lessa et al., 2012; Dumitru et al., 2017). Like other infections, CDI is primarily treated with antibiotics. Only three antibiotics, including metronidazole, vancomycin and fidaxomicin, are currently available to treat CDI, each of which faces challenges with their use (Morado and Nanda, 2025). Specifically, metronidazole and vancomycin cause significant collateral damage to the gut microbiota, which predisposes patients to high rates of recurrent infection (Shin and Warren, 2017). Furthermore, while fidaxomicin is more microbiome-sparing, its widespread use is often limited by high cost (Jiang et al., 2022). Therefore, it is necessary to continue developing effective and novel therapies that are microbiome friendly to treat and prevent CDI.

Despite this urgent need, the drug development process tends to be costly and time consuming, beginning with the development and use of *in vivo* pre-clinical models. Although rodents usually are commonly used, their care and use is often expensive and ethically challenging (Graham and Prescott, 2015). As a result, more biologically simple invertebrate *in vivo* models have begun to partially replace or reduce the number of vertebrates used. *Caenorhabditis elegans* has been utilized to study human pathogens, but several limitations exist with this model that are resolved with the use of the waxworm larvae, *G. mellonella* (Loh et al., 2013; Champion et al., 2016). First, *Galleria mellonella* larvae can tolerate human body temperatures (37 °C), which make it ideal to study human pathogens under physiologically accurate conditions. Additionally, *G. mellonella* has a basic immune response that can be utilized to assess host response (Kavanagh and Reeves, 2004). Furthermore, this model is responsive to antibiotic treatment and can be used to assess the efficacy of new antimicrobial agents (Aperis et al., 2007; Mylonakis et al., 2005). Finally, *G. mellonella* larvae are inexpensive, easy to handle, and have short life cycles and fewer ethical considerations associated with their use (Champion et al., 2016).

Due to these advantages, a *G. mellonella* larvae model has been utilized to study the pathogenesis of several human pathogens including *Staphylococcus aureus* (Sheehan et al., 2019), *Streptococcus pneumoniae* (Cools et al., 2019), *Klebsiella pneumoniae* (Insua et al., 2013), *Campylobacter jejuni* (Senior et al., 2011), *Listeria monocytogenes* (Joyce and Gahan, 2010), and anaerobic bacteria including *Clostridium perfringens* and *C. difficile* (Nale et al., 2016; Nale et al., 2020; Kay et al., 2019). While several studies have successfully employed *G. mellonella* to investigate CDI, much of this previous work has focused on specific therapeutic applications, such as evaluating phage therapy efficacy (Nale et al., 2016; Nale et al., 2020). Furthermore, these earlier investigations often utilized a limited number of *C. difficile* strains and did not systematically compare a wide array of toxigenic versus non-toxigenic clinical ribotypes. Consequently, a broadly validated, standardized oral gavage model that compares diverse, clinically significant ribotypes and establishes clear baseline parameters for evaluating standard-of-care antibiotics remains absent from the literature. Therefore, this study utilized *G. mellonella* to address this gap by developing a standardized oral infection model, comprehensively evaluating pathogenesis across a diverse strain panel, and assessing therapeutic outcomes using vancomycin as a standard-of-care control.

## Methods

### Development and optimization of the model

*Bacterial Strains and* Inoculum *Preparation*: Standard *C. difficile* reference strains were purchased from ATCC (American Type of Culture Collection, Manassas, Virginia), while clinical *C. difficile* isolates were obtained from an active surveillance system (Gonzales-Luna et al., 2020). Due to the de-identified nature of this repository, specific patient-level clinical data (e.g., disease severity, demographics, or clinical status) associated with these isolates were not available. The presence of toxin genes was determined using multiplex PCR (Aitken et al., 2015), and fluorescent PCR ribotyping was performed as previously described (Alam et al., 2017). All selected isolates were previously characterized and confirmed to be susceptible to vancomycin (MIC ≤2 ug/mL) prior to *in vivo* testing. A full list of the *C. difficile* isolates used is shown in Table 1. Isolates were extracted from stool samples and cultured anaerobically as described previously (Edwards et al., 2013).

**TABLE 1:** List of *C. difficile* isolates used in the study.

Strain	Ribotype	Source of strain
MT-5368	014/020	Clinical
MT-5369	014/020	Clinical
MT-5039	014/020	Clinical
MT-5479	F027	Clinical
MT-5430	F027	Clinical
MT-5466	F027	Clinical
R20291	F027	Reference strain
MT-5489	F106	Clinical
MT-5421	F106	Clinical
MT-5356	F106	Clinical
CD630	F012	Reference strain
ATCC 700057	F038	Reference strain

To mimic the natural, transmissible form of the pathogen upon oral ingestion, all experiments utilized *C. difficile* spores. Bacterial cultures containing *C. difficile* spores for larval infection were prepared as previously described (Edwards et al., 2013). Briefly, spores were generated by inoculating a single colony of each isolate onto blood agar and incubating in an anaerobic chamber for 3 days. Colonies were transferred to microcentrifuge tubes containing sterile water and heated for 30 min at 70 °C to eliminate remaining vegetative cells. The bacterial suspension was centrifuged at 5,000 *g* for 3 min, and the pellet was re-suspended in an equal volume of sterile water. This washing step was repeated three times to remove growth residues, and the purified spore stock was stored at 4 °C until use.

To accurately standardize the inoculum, the purified spore stock was serially diluted in sterile water and plated onto pre-reduced blood agar. Following anaerobic incubation, colonies were enumerated to determine the exact spore titer. Based on these calculations, the stock was subsequently diluted to achieve the precise target working concentration. The final bacterial spore suspension of 1 × 10^7^ CFU/mL was then retrospectively confirmed by manual colony forming unit (CFU) count immediately prior to gavage.

**FIGURE 1:**
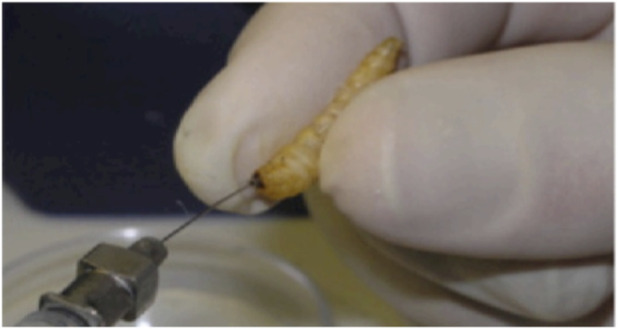
Gavaging of *Galleria mellonella*.

*G*. *mellonella* preparation and storage: Last-instar *G. mellonella* larvae were obtained from a commercial supplier (Timberline, USA) and stored in wood shavings at 15 °C. Larvae were not fed prior to or during experiments. Prior to use, larvae were acclimatized at 30 °C for several hours. Only healthy larvae weighing between 250 and 350 mg were selected for experiments. Bacterial inoculation was performed via oral gavage. Larvae were gently immobilized manually between the investigator’s fingers; to prevent cold-shock and maintain their prior acclimatization at 30 °C, larvae were not chilled prior to the procedure. A precise volume of 10 μL of the bacterial suspension (or phosphate-buffered saline [PBS] control) was administered directly into the larval foregut using a U-100 insulin syringe (UltiCare, USA) (Figure 1). Post-inoculation, the larvae were placed in sterile Petri dishes and observed for 10 min to verify delivery and ensure the inoculum was fully retained without regurgitation or leakage. Any larvae exhibiting leakage or immediate physical trauma were discarded and replaced. To validate the model, all experiments included two control groups: a PBS-gavaged group was included to control for the physical trauma of the gavaging procedure and ensure the vehicle itself did not contribute to mortality, and an unmanipulated control group was maintained to assess the baseline health and viability of the larvae batch. Each experimental arm consisted of ten larvae, and all experiments were performed in duplicate. Survival was monitored as a binary outcome The progression of infection was visually tracked via the melanization process, which is characterized by a gradual shift from a healthy cream/beige color to widespread dark pigmentation as a result of the insect’s humoral immune response. Larvae were classified as dead if they were unresponsive to external stimuli or exhibited complete melanization. Experiments were discarded and repeated if mortality exceeded two larvae in either control group. For clarity, control data are excluded from the figures.

Pre-incubation duration assessment: To evaluate the impact of larval age on survival outcomes, killing assays were conducted using *G. mellonella* larvae stored in wood shavings at 15 °C for either <7 days (“fresh”) or ≥7 days (“old”) prior to use. Larvae were inoculated with 10 μL of either phosphate-buffered saline (PBS) as a control or a suspension of toxigenic *C. difficile* (strain MT-5369/RT 014/020) at an inoculum of 1 × 10^7^ CFU/mL. Following inoculation, larvae were incubated at 37 °C, and survival was monitored daily for 120 h.

Optimal growth temperature determination: To determine the optimal temperature for *C. difficile* infection in this model, killing assays were conducted at 30 °C and 37 °C using a range of bacterial inocula (1 × 10^3^–1 × 10^7^ CFU/mL). These specific temperatures were selected to compare the standard physiological optimal temperature for *G. mellonella* health and immune function (30 °C) against human core body temperature (37 °C). Testing at 37 °C is critical to accurately simulate the clinical host environment and stimulate physiologically relevant bacterial pathogenesis and toxin production. These assays evaluated both toxigenic (MT-5369/RT 014/020) and non-toxigenic (ATCC 700057/RT 038) *C. difficile* strains.

Median lethal dose (LD50) determination: Killing assays were conducted to identify the optimal bacterial inoculum for studying CDI pathogenesis and treatment. *G. mellonella* larvae were gavaged with the toxigenic strain MT-5369 (RT 014/020) using a range of bacterial inocula (1 × 10^5^–1 × 10^9^ CFU/mL). Post-inoculation, larvae were incubated at 37 °C, and survival was monitored daily for 120 h. The median LD50 was calculated and subsequently used for model validation experiments (data not shown).

### Model validation and analysis

*C*. *difficile* RT virulence: To evaluate the utility of the *G. mellonella* model for virulence studies, larvae were infected with various clinical and reference *C. difficile* strains at a standardized inoculum of 1 × 10^7^ CFU/mL. The panel of toxigenic strains included RT027, RT106, RT014/020, and RT012 (Table 1). To determine the specific impact of toxin production on larval survival, the non-toxigenic reference strain (ATCC 700057/RT 038) was compared against the toxigenic strain MT-5369 (RT 014/020), which served as the primary test strain during the model optimization phase.

Antibiotic efficacy: To evaluate the feasibility of using *G. mellonella* larvae to study new CDI antimicrobials, larvae were infected with *C. difficile* MT-5369. This strain was selected to maintain consistency with the model optimization experiments described above. Larvae were gavaged with varying concentrations of vancomycin (2, 0.2, 0.02, 0.002, and 0.0002 mg/kg). This logarithmic dosing range was adapted from previous efficacy studies (Nale et al., 2016; Nale et al., 2020) to establish a minimum effective dose and dose-response curve for this model.

Antibiotic Preparation and Administration: Vancomycin was purchased from Sigma Pharmaceuticals (North Liberty, Iowa) and prepared according to the package insert. To model infection and treatment, larvae in each group were subjected to two sequential gavage steps. First, larvae were inoculated with the bacterial suspension (or PBS vehicle) and incubated at 37 °C for 2 h to establish infection. Following this incubation, a second gavage was administered containing the assigned vancomycin dose or PBS control. The experimental groups were defined as follows: (1) Treatment group (bacteria followed by antibiotic), (2) Infection control (bacteria followed by PBS to monitor untreated disease progression), and (3) Toxicity control (PBS followed by antibiotic to confirm that the antibiotic dose itself was not lethal to the larvae). Prior to the main efficacy trials, toxicity testing using the maximum vancomycin dose (2 mg/kg) was performed to ensure safety in uninfected larvae. Post-treatment, larvae were incubated and survival was monitored as described previously. To corroborate survival data, bacterial burden was quantified using quantitative PCR (qPCR) 24 h after treatment.

Bacterial DNA Extraction and Quantification: Total DNA was extracted from individual *G. mellonella* larvae to quantify *C. difficile* burden. Larvae were homogenized in 1 mL of sterile water. To control for extraction efficiency and potential PCR inhibition, 200 μL of a suspension containing heat-killed *Escherichia coli* was added to each sample as an internal spike-in control prior to extraction. The homogenate was vortexed for 10–20 s and centrifuged at 10,000 rpm for 3 min to pellet debris. DNA was purified from the supernatant and eluted in a final volume of 50 μL. DNA quality and concentration were assessed using a Qubit 4 Fluorometer (ThermoFisher Scientific), A260/A280 absorbance ratios, and 1% agarose gel electrophoresis.

Quantitative PCR (qPCR) Conditions: Quantification of *C. difficile* load was performed using species-specific primers targeting the tcdB gene (Forward: 5′-GGG​AGC​TTC​CCA​TAC​GGG​TTG-3’; Reverse: 5′-TTG​ACT​GCC​TCA​ATG​CTT​GGG​C-3′), yielding a 300 bp amplicon. Reactions were carried out in triplicate in a 20 μL final volume containing 10 μL of 2x QuantiTect SYBR Green PCR Master Mix (Qiagen), 0.5 μM of each primer, and 5 μL of template DNA.

Real-time PCR was performed on a QuantStudio™ 5 System (Applied Biosystems) with the following thermal cycling conditions: initial activation at [95 °C for 15 min], followed by 40 cycles of denaturation at [94 °C for 15 s], annealing at [55 °C for 30 s], and extension at [72 °C for 30 s]. To verify the specificity of the amplification, a melt curve analysis was performed immediately following the PCR cycles (60 °C–95 °C, increasing by 0.15 °C/s).

Standard Curve and Data Analysis: Bacterial load was quantified using a standard curve generated from TOPO-cloned *C. difficile* PCR products (Invitrogen). The plasmid insert was verified by Sanger sequencing and BLAST analysis. A ten-fold serial dilution series of the plasmid standard (5 × 10^8^ to 500 copies) was included on each plate. The standard curve exhibited a linear range with an amplification efficiency between [90% and 110%] and an *R* ^*2*^ value > [0.99]. Threshold cycle (*Ct*) values were converted to genome copies per larva, and results were expressed as the change in bacterial load (Δ log_10_ copies/larva) relative to the initial inoculum.

### Statistical analysis

Survival curves were generated using the Kaplan-Meier method, and differences in survival were assessed using the log-rank test. All analyses were performed using STATA 16 software, with statistical significance defined as a P-value ≤0.05.

Ethical Approval: Ethical approval was not required for this study. All *in vivo* infection and treatment experiments were conducted using *G. mellonella*, an invertebrate wax moth larva. Invertebrate models do not fall under the regulatory purview of Institutional Animal Care and Use Committees (IACUC) or equivalent animal ethics boards, and thus are exempt from formal ethical approval requirements.

## Results

### Use of fresh *Galleria mellonella* larvae improves survival assessment

**FIGURE 2:**
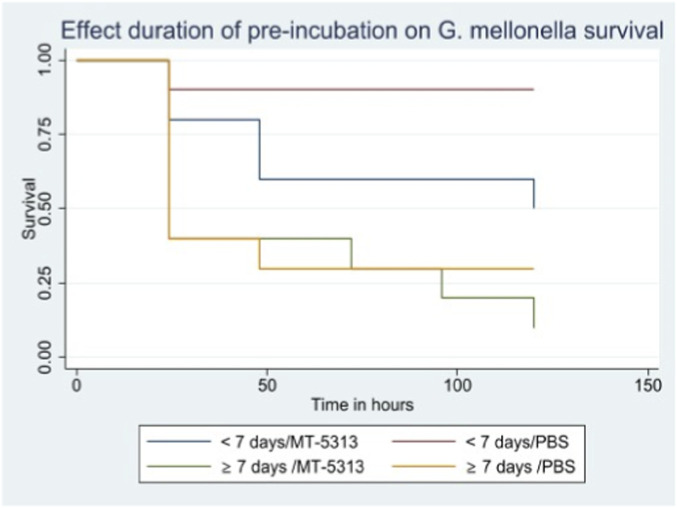
Effect duration of pre-incubation on *Galleria mellonella* survival.

Larvae stored for ≥7 days at 15 °C exhibited significantly lower survival compared to fresh larvae (<7 days) (P = 0.003) (Figure 2). Among fresh larvae infected with 1 × 10^7^ CFU/mL, 50% (10/20) survived, compared to 90% (18/20) in the PBS control group. In contrast, survival in older larvae (≥7 days) dropped to 10% (2/20) in the infected group and 30% (6/20) in the control group.

### Survival of *Galleria mellonella* larvae is maintained at human physiological temperature

**FIGURE 3:**
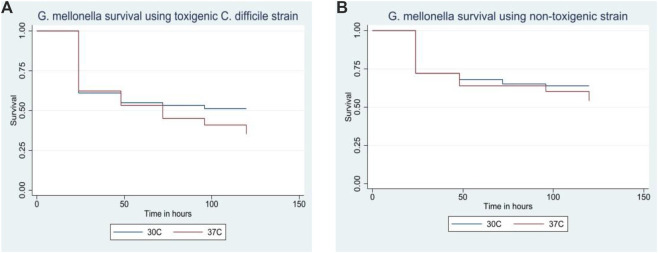
**(A)** Effect of temperature on *Galleria mellonella* survival using toxigenic strain/ribotype (MT-5369/RT 014/020). **(B)** Effect of temperature on *Galleria mellonella* survival using non-toxigenic strain/ribotype (ATCC 700057/RT038).

Infection with 1 × 10^7^ CFU/mL of the toxigenic *C. difficile* strain MT-5369 resulted in 49% (49/100) survival at 30 °C, compared to 65% (65/100) at 37 °C (P = 0.05) (Figure 3A). Although this difference was not statistically significant, we proceeded with experiments at 37 °C to better simulate the human physiological conditions required for *C. difficile* pathogenesis. Similarly, temperature did not significantly impact survival in larvae infected with the non-toxigenic ATCC 700057 strain (36% [36/100] at 30 °C vs. 46% [46/100] at 37 °C, P = 0.2) (Figure 3B).

### An inoculum size of 1 × 10^7^ CFU/mL was optimal to study CDI pathogenesis and treatment

**FIGURE 4:**
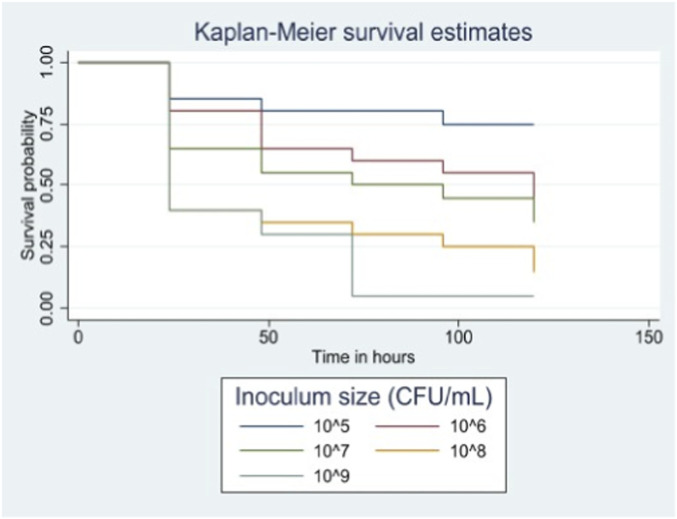
Effect of inoculum size on *Galleria mellonella* survival.

Survival rates were inversely proportional to the bacterial load, ranging from 5% (1/20) at the highest concentration (1 × 10^9^ CFU/mL; 1 × 10^7^ CFU/larva) to 75% (15/20) at the lowest (1 × 10^5^ CFU/mL; 1 × 10^3^ CFU/larva) (Figure 4). An intermediate inoculum of 1 × 10^7^ CFU/mL (1 × 10^5^ CFU/larva) resulted in 40% survival (8/20) at 37 °C. This inoculum was selected for subsequent experiments as it provided an optimal baseline mortality (∼60%) to evaluate both therapeutic efficacy (rescue) and virulence factors.

### Survival of *Galleria mellonella* larvae is dependent on *C. difficile* ribotype and toxin production

**FIGURE 5:**
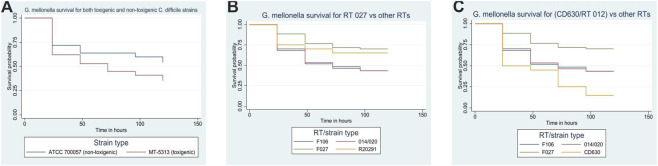
**(A)** Comparison of *Galleria mellonella* survival between toxigenic (MT-5369/RT 014/020) and non-toxigenic (ATCC 700057/RT 038) strains. **(B)** Comparison of *Galleria mellonella* survival between the reference strain R20291/RT 027 and other strains/RTs. **(C)** Comparison of *Galleria mellonella* survival between the reference strain CD630/RT 012 and other strains/RTs.

Overall, survival was significantly lower in larvae infected with the toxigenic strain MT-5369 compared to the non-toxigenic strain ATCC 700057 (35% [35/100] vs. 54% [54/100], respectively; P = 0.003) (Figure 5A). We also observed significant variation in virulence among clinical ribotypes. Survival in larvae infected with RT 106 (43%; 26/60) and RT 014/020 (43%; 26/60) was significantly lower than in those infected with the reference strain R20291/RT 027 (65%; 13/20) (P = 0.003) (Figure 5B). Furthermore, the reference toxigenic strain CD630/RT 012 exhibited the highest virulence, resulting in significantly lower survival (15%; 3/20) compared to all other tested ribotypes (P < 0.0001) (Figure 5C).

### Vancomycin treatment improved survival of *Galleria mellonella* larvae and decreased *C. difficile* bacterial burden

**FIGURE 6:**
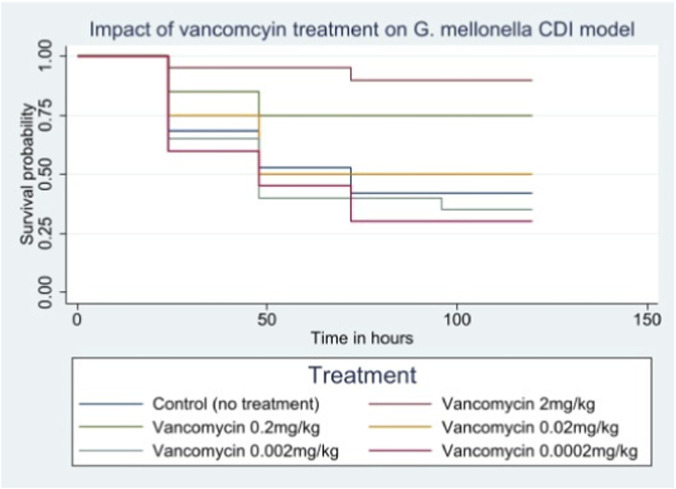
Comparison of *Galleria mellonella* survival among different vancomycin dosing regimens.

**FIGURE 7:**
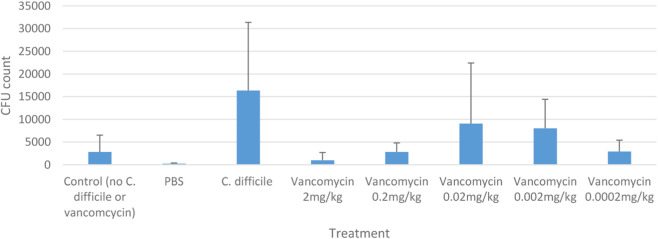
Comparison of *C. difficile* burden in *Galleria mellonella* larvae in different treatment options.

Among the five dosing regimens investigated, the highest vancomycin concentration (2 mg/kg) yielded the highest survival rate (90%; 18/20). Survival was significantly higher in groups treated with ≥0.2 mg/kg compared to untreated controls (P < 0.001). Conversely, doses ≤0.02 mg/kg resulted in survival rates statistically indistinguishable from the control group (Figure 6). To assess bacterial persistence, *C. difficile* burden was quantified via qPCR. Consistent with the survival data, the most significant reduction in bacterial load was observed at the maximum vancomycin dose (2 mg/kg). In contrast, bacterial loads in larvae treated with ≤0.02 mg/kg remained high, comparable to the untreated control group (Figure 7). Overall, the reduction in bacterial burden broadly corresponded with the increased survival rates observed in the high-dose treatment groups.

## Discussion

The adoption of surrogate *in vivo* models, such as *G. mellonella* larvae, represents a pivotal step in facilitating drug development for infectious diseases. While the hamster model remains the standard for CDI studies, its utility is constrained by high maintenance costs, rigorous regulatory requirements, and limited sample sizes allowed per experiment (Best et al., 2012). Furthermore, hamsters exhibit hypersensitivity to *C. difficile* toxins, often resulting in disproportionately high mortality rates that may not accurately reflect human clinical outcomes (Buckley et al., 2013). *G. mellonella* offers distinct advantages over mammalian models regarding cost and ease of use. Moreover, it possesses advantages over other *in vivo* invertebrate models, as the larvae possess cellular and humoral antimicrobial defenses comparable to the mammalian innate immune system, making them highly suitable for assessing infection and therapeutic interventions (Kay et al., 2019; Vogel et al., 2011). Although *G. mellonella* has previously been utilized in limited capacities to study CDI bacterial burden and specific treatments (Nale et al., 2016; Nale et al., 2020), no universally standardized protocol has been established. To our knowledge, this report is the first to present a comprehensively optimized oral gavage model—systematically defining crucial baseline parameters including optimal storage, temperature, and exact spore inoculum—and the first to validate this platform across a diverse panel of clinically significant *C. difficile* strains. Our findings elevate the utility of *G. mellonella* from an *ad hoc* experimental tool to a robust, highly reproducible, high-throughput model for investigating CDI pathogenesis and screening novel compounds via survival analysis.

During model optimization, we observed that *G. mellonella* susceptibility significantly increased if larvae were subjected to prolonged cold storage (>7 days) prior to infection, a finding consistent with previous reports (Browne et al., 2015). For example, in *S. aureus* infection models, larval survival decreased from 65.5% after 1 week of storage to 13.3% after 10 weeks (P < 0.001) (Browne et al., 2015). Although our study evaluated a much shorter interval (7 days vs. 10 weeks), a similar decline in robustness was evident. To minimize the confounding variable of larval age, we established a strict quality control criterion: larvae were utilized for experiments within 7 days of receipt from the vendor and discarded thereafter.

Our study confirmed an optimal *C. difficile* inoculum of 1 × 10^7^ CFU/mL (1 × 10^5^ CFU/larva) at 37 °C. This aligns with findings by Browne et al., who first investigated the inoculum effect in *G. mellonella* and determined that a dose of 10^5^ CFU/larva was optimal for studying CDI therapies (Browne et al., 2015). Regarding temperature, Peleg et al. demonstrated that *G. mellonella* survival is typically lower when incubated at 37 °C post-infection compared to 30 °C (P = 0.01) (Peleg et al., 2009). In contrast, we did not observe a significant difference in survival between these temperatures. Although survival was numerically higher at 37 °C in our model, this difference did not reach statistical significance. These data suggest that the virulence of *C. difficile* in *G. mellonella* larvae may not be directly related to the temperature of incubation, or that our study was underpowered to detect a difference if one exists.

Our data further demonstrated a clear correlation between *C. difficile* toxin production and *G. mellonella* mortality. This is consistent with a previous report by Abuderman et al., which showed that toxigenic *C. difficile* strains were significantly more virulent than non-toxigenic ones (Abuderman et al., 2018). Additionally, we observed higher virulence in clinical isolates compared to the reference strain R20291 (RT 027). Interestingly, the historic reference strain CD630 (RT 012) exhibited high virulence in this model, comparable to or exceeding that of the clinical isolates. This stands in contrast to murine models, where R20291 is typically hypervirulent and CD630 is considered less pathogenic (Li et al., 2018). While significant differences in survival were observed *between* different *C. difficile* ribotypes, survival outcomes remained consistent among different strains *within* the same ribotype.

The discordance in virulence observed between reference strains and clinical isolates in our study highlights the complexity of modeling host-pathogen interactions. Similar variability has been documented in other bacterial species; for instance, Peleg et al. reported that community-acquired *Acinetobacter baumannii* strains exhibited greater lethality in *G. mellonella* than hospital-acquired reference strains (Peleg et al., 2009). Conversely, McLaughlin et al. found a discordance in virulence between human outcomes and *G. mellonella* survival when studying KPC-producing *K. pneumoniae* (McLaughlin et al., 2014). This variation may be partially attributed to the absence of an adaptive immune system in *G. mellonella* larvae (Briles et al., 1992). However, it is crucial to note that mammalian models also possess inherent limitations in predicting human pathogenicity. Briles et al., for example, demonstrated that *S. pneumoniae* serotype 19F, which is virulent in humans, lacked virulence in murine models (Briles et al., 1992). Therefore, while *G. mellonella* may not perfectly mirror the adaptive immune response of higher mammals, it serves as a valuable, high-throughput surrogate for identifying virulence factors and screening antimicrobial efficacy prior to mammalian testing.

To validate the predictive utility of our model, we assessed the efficacy of vancomycin, the standard-of-care antibiotic for CDI. We demonstrated a clear dose-dependent survival benefit with vancomycin doses ≥0.2 mg/kg, accompanied by a significant reduction in bacterial burden (Figure 7), consistent with previous reports (Nale et al., 2016; Nale et al., 2020). Although the statistical separation in bacterial load was less distinct than in the survival analysis, this is likely attributable to the nature of qPCR quantification. Unlike culture-based methods used in previous studies, qPCR detects DNA from both viable and non-viable cells. Consequently, the persistence of DNA from bacteria killed by vancomycin may dampen the observed statistical difference, even when the therapeutic effect on survival is profound. We selected this molecular approach to avoid the potential subjectivity of manual counting and to improve the limit of detection. While trace amplification signals were detected in the effective treatment groups, these levels were negligible compared to the high bacterial burden in untreated infected larvae. These low-level signals likely represent the limit of quantification or detection of residual non-viable DNA rather than active infection. Overall, these data indicate that *G. mellonella* serves as a robust infection model for screening new antimicrobial agents, offering a high-throughput, cost-effective alternative to mammalian systems.

A primary strength of this investigation is the comprehensive standardization of the *G. mellonella* model for CDI. We systematically optimized critical variables—including temperature, cold storage duration, and inoculum size—and validated these parameters against a diverse panel of clinical and reference *C. difficile* strains. Furthermore, the inclusion of robust positive and negative control arms ensured the reproducibility of our findings.

However, our study has limitations warranting discussion. First, to maximize the model’s accessibility, we utilized standard insulin syringes for gavage rather than the automated Hamilton syringe pumps employed in previous studies (Nale et al., 2016; Nale et al., 2020). While manual dosing lowers the technical barrier for adoption, it may introduce minor variability compared to automated systems. Additionally, while our sample size (n = 10 per group, performed in duplicate) adheres to standard practices for invertebrate models and was sufficient to achieve statistical significance, larger sample sizes in future studies could further refine the precision of survival estimates. Second, our primary endpoints were restricted to larval survival and bacterial burden. Future investigations should incorporate host gene expression analysis and immune response profiling to fully characterize the host-pathogen interaction.

Additionally, *in vivo* imaging (bioluminescence) would provide valuable spatiotemporal data, as previously described (Joyce and Gahan, 2010; Delarze et al., 2015). Furthermore, while our qPCR approach successfully quantified the burden of toxigenic bacteria, direct quantitative measurement of *C. difficile* exotoxin (TcdA and TcdB) accumulation within the larval hemolymph was not performed. Incorporating direct toxin assays during infection and post-treatment in future investigations would offer deeper mechanistic insights into both pathogenesis and therapeutic neutralization (Nale et al., 2020). Finally, we utilized commercially sourced larvae from a standard vendor rather than research-grade stock reared in-house. While this introduces potential variability, it demonstrates the model’s robustness and ensures that this protocol is easily reproducible by laboratories without specialized insect-rearing facilities.

## Conclusion

*G. mellonella* has emerged as a vital *in vivo* platform for infectious disease research, offering a rapid and scalable alternative to traditional animal models. Our findings validate the utility of *G. mellonella* larvae as a robust pre-clinical screen for evaluating novel CDI therapeutics. While this model serves as an effective bridge between *in vitro* assays and mammalian studies, it functions as a complementary screening tool rather than a replacement for vertebrate models. Crucially, researchers must recognize that virulence patterns of specific *C. difficile* ribotypes in larvae may diverge from those observed in murine or hamster models, underscoring the distinct host-pathogen interactions inherent to invertebrate systems. Nevertheless, this high-throughput platform offers a cost-effective and ethically simplified avenue for early-stage pharmacokinetic and pharmacodynamic (PK/PD) profiling. Widespread adoption of this standardized protocol has the potential to accelerate the drug discovery pipeline for *C. difficile* infection by identifying promising candidates prior to mammalian testing.

## Data Availability

The raw data supporting the conclusions of this article will be made available by the authors, without undue reservation.
